# Enhanced myostatin expression and signalling promote tubulointerstitial inflammation in diabetic nephropathy

**DOI:** 10.1038/s41598-020-62875-2

**Published:** 2020-04-14

**Authors:** Daniela Verzola, Samantha Milanesi, Francesca Viazzi, Francesca Ansaldo, Michela Saio, Silvano Garibaldi, Annalisa Carta, Francesca Costigliolo, Gennaro Salvidio, Chiara Barisione, Pasquale Esposito, Giacomo Garibotto, Daniela Picciotto

**Affiliations:** 1 Division of Nephrology, Dialysis and Transplantation, University of Genova, Department of Internal Medicine and IRCCS Ospedale Policlinico San Martino, Genova, Italy; 2 Division of Cardiology, University of Genova, Department of Internal Medicine and IRCCS Ospedale Policlinico San Martino, Genova, Italy

**Keywords:** Translational research, Diabetic nephropathy

## Abstract

Myostatin (MSTN), a family member of the transforming growth factor (TGF)-β super family, has been detected in the tubuli of pig kidney, but its role in the human kidney is not known. In this study we observed upregulation of MSTN mRNA (~8 to 10-fold increase) both in the glomeruli and tubulointerstitium in diabetic nephropathy (DN). In DN, immunoreactive MSTN was mainly localized in the tubuli and interstitium (∼4–8 fold increase), where it colocalized in CD45^+^ cells. MSTN was also upregulated in the glomeruli and the arterial vessels. Tubulointerstitial MSTN expression was directly related to interstitial fibrosis (r = 0.54, p < 0.01). In HK-2 tubular epithelial cells, both high (30 mmol) glucose and glycated albumin upregulated MSTN mRNA and its protein (p < 0.05–0.01). MSTN-treated HK-2 cells underwent decreased proliferation, together with NF-kB activation and CCL-2 and SMAD 2,3 overexpression. In addition, MSTN induced intracellular ROS release and upregulated NADPH oxidase, effects which were mediated by ERK activation. In conclusion, our data show that MSTN is expressed in the human kidney and overexpressed in DN, mainly in the tubulointerstitial compartment. Our results also show that MSTN is a strong inducer of proximal tubule activation and suggest that MSTN overexpression contributes to kidney interstitial fibrosis in DN.

## Introduction

Myostatin (MSTN), a family member of the transforming growth factor (TGF)-β super family, is a major effector of muscle atrophy in several chronic diseases, including chronic kidney disease (CKD)^1–5^. MSTN has many similarities to TGF-β in structure, signaling pathway and functions^4,5^. After the cleavage of its mature COOH-terminus domain, MSTN binds to the activin-type II receptor B (Act RIIB), and to activin-type II receptor A (Act RIIA) with lower affinity, while recruiting both Activin-type I receptors, ALK4 and ALK5^1,6^. The binding of MSTN and activin to the Act RIIA/B receptor complex activates SMAD2,3-mediated transcription, which accelerates protein breakdown and inhibits protein synthesis^6,7^. By activating SMAD2,3 MSTN also causes fibrosis, since it stimulates fibroblast proliferation and induces its differentiation into myofibroblasts. In addition, MSTN shares with TGF-β1 a positive feed-back loop, with TGF-β1 stimulating MSTN expression, and, conversely, MSTN inducing TGF-β1 secretion^8^.

MSTN expression is not only limited to skeletal muscle; low levels of MSTN mRNA are reported in several other types of human tissues, including smooth muscle, cardiac muscle, adipose tissue, the brain, the spleen and circulating leucocytes^1^. MSTN has also been detected in the tubular compartment of the kidney of the pig^9^ a species that is anatomically and physiologically comparable to humans^10^, suggesting that MSTN may have functions also in the human kidney.

In addition to its influences on protein metabolism, MSTN has many effects on glucose metabolism, and may participate in the pathophysiology of diabetes mellitus. MSTN deficiency protects against high-fat diet-induced obesity^11^, tissue inflammation^12^, insulin resistance^13,14^ and atherosclerosis^15^. MSTN inhibition leads to increased insulin sensitivity^16^. The involvement of MSTN in diabetes is suggested by elevated MSTN mRNA levels in skeletal muscle biopsies of obese, insulin–resistant subjects^17^ and from non-obese, hyperinsulinemic relatives of patients with type 2 diabetes (T2DM)^18^. In extreme obesity and T2DM, MSTN is positively associated with fasting plasma glucose and “static” indexes of insulin resistance^19^. In addition, other data show that in aortic atherosclerosis, MSTN is upregulated in the media, neointima and in cells infiltrating the vessel wall, where it plays an active role in monocyte chemiotaxis and ultimately, in vascular wall remodeling^20^.

Despite the abnormal MSTN regulation in insulin resistance, atherosclerosis, obesity and diabetes, the possible direct role of MSTN in diabetic nephropathy (DN) is unknown. In the current study, we tested the hypothesis that a MSTN-dependent inflammatory response is enhanced in DN. We tested this postulate with different selected measures. First, we evaluated the MSTN gene and protein expression in the normal kidney and in DN. In the diabetic kidney we observed overexpression of MSTN in the glomeruli and tubulointerstitium, which correlated with glomerulosclerosis and interstitial fibrosis, respectively. Second, in HK-2 cells we tested the effects of MSTN on its downward pathways in muscle and we observed that MSTN slows cell proliferation, activates NF-kB activation and upregulates SMAD 2,3 and fibronectin production. In addition, MSTN induces intracellular Reactive Oxygen Species (ROS) release via upregulation of NADPH oxidase and ERK activation. Third, we observed that the diabetic milieu upregulates MSTN in proximal tubule cells (HK-2). The results obtained in this study indicate that MSTN is expressed in the human kidney and overexpressed in DN, and suggest that MSTN plays a role in tubulointerstitial inflammation and fibrosis.

## Results

### MSTN is expressed in the normal kidney and is upregulated both in the glomeruli and in the tubulointerstitium of patients with DN

**Figure 1:**
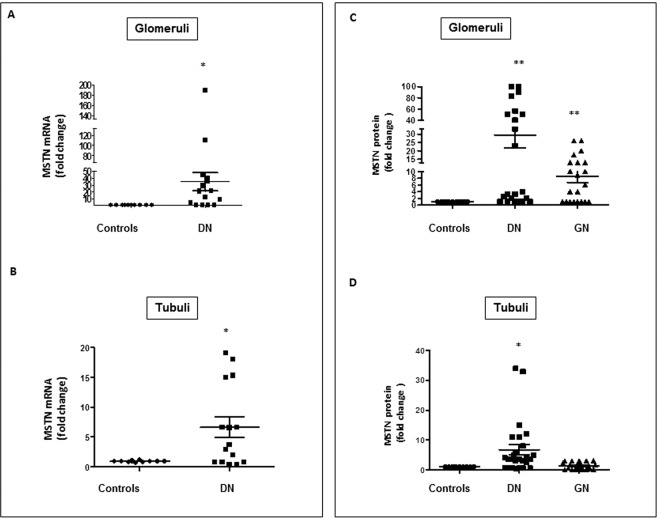
Expression of MSTN mRNA and protein in the glomeruli and tubulointerstitium of controls (controls) (n = 13) and patients with DN (n = 16). (**A,B**) mRNA levels of MSTN were determined by rt-PCR in laser microdissected glomeruli and tubulointerstitium. (**C,D**). Glomerular and tubular protein expression of MSTN was evaluated by immunohistochemistry and the positive area by image analysis in controls (N = 13), DN (N = 26), and patients with IgA nephropathy (n = 11) or FSGS (n = 11). Results are expressed as fold change ±SEM to the controls (*p < 0.05, **p < 0.01 vs controls). MSTN = Myostatin, DN = Diabetic nephropathy, IgA = immunoglobulin A nephropathy, FSGS = focal segmental glomerulosclerosis, Rt-PCR = real time PCR.

**Figure 2:**
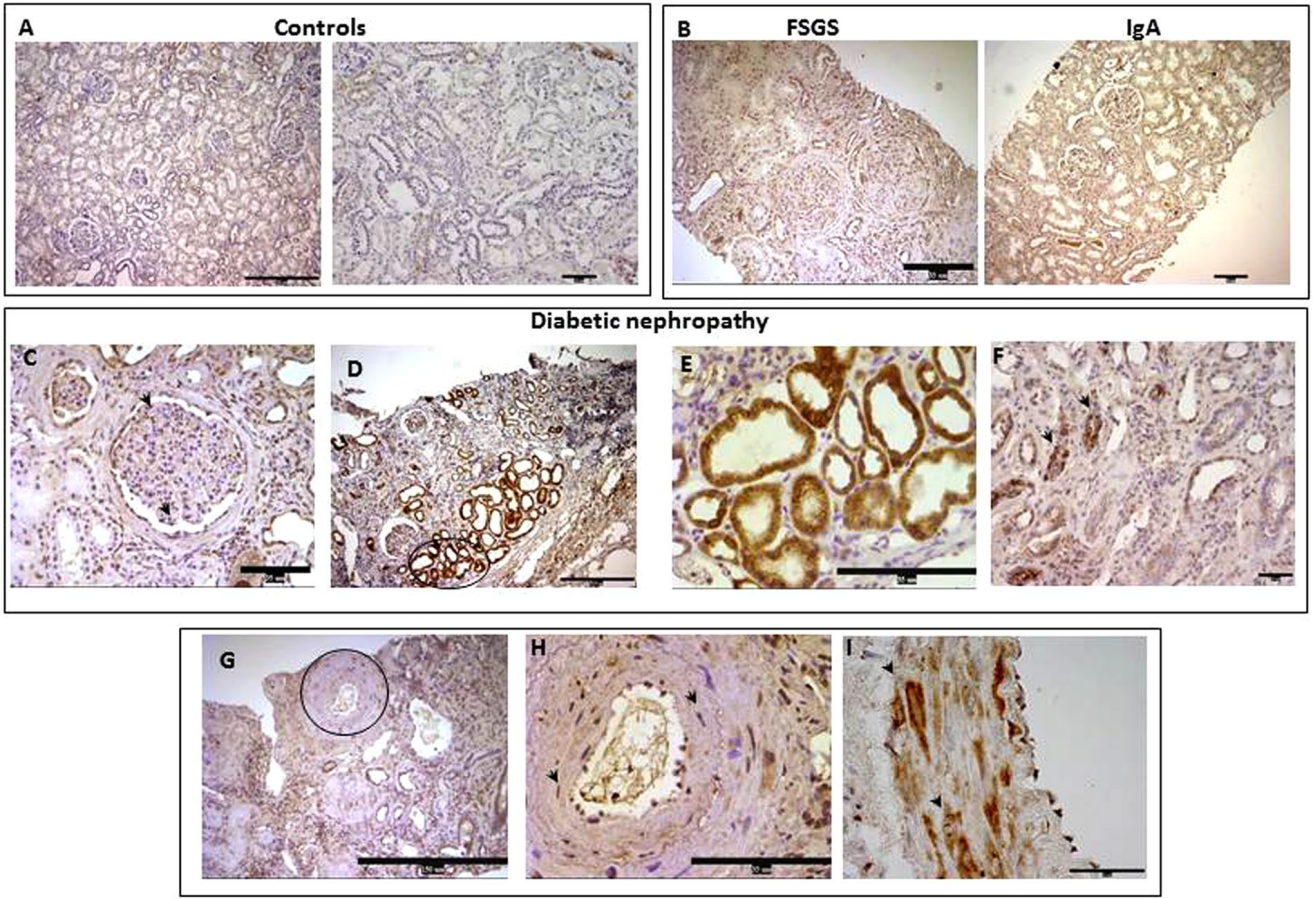
MSTN in human renal biopsies evaluated by immunohistochemistry. Pictures are representative of MSTN expression in controls (**A**), IgA and FSGS (**B**), and DN (**C–I**). MSTN was absent or very faintly expressed in the tubular compartment of both control (**A**) and in IgA/FSGS tubuli (**B**), while it was detectable in the glomeruli of IgA/FSGS patients (**B**). In DN, MSTN was detected in glomerular cells, such as podocytes (arrows) (**C**) and in cytoplasm of tubule cells (**D–F**). In addition, MSTN was detected in the vessel walls (**G**), in particularly in VSMC cytoplasm (**H**,**I**). Arrows indicate positive cells and the round box highlights the image depicted in **H**. MSTN = Myostatin, IgA = Immunoglobulin A nephropathy, FSGS = focal segmental glomerulosclerosis; DN = diabetic nephropathy; P = Podocytes; VSMCs = Vascular smooth muscle cells. Magnification: x100 (Bar = 150 µM), panel A left figure, (**D,G**); x200 (Bar = 35 µM), panel A right figure, Panel B; x400 (Bar = 35 µM) (**C**,**E**,**F**,**H**); X1000 (Bar = 15 µM), I.

Figure 1A,B shows MSTN mRNA levels in the glomerular and tubulointerstitial compartments of microdissected biopsies of normal controls and DN. In the normal kidney MSTN mRNA was expressed at low level in both in the tubular and glomerular compartments and its protein was very faintly expressed in the tubuli and interstitium (Figs. 1C,D and 2A). MSTN mRNA was markedly overexpressed in DN in the glomeruli and tubulointerstitium (a ~20 to 40-fold increase vs. controls, and a ~4 to 8-fold increase vs. glomerular diseases, respectively, p < 0–05–0.01). In addition, MSTN protein was upregulated in DN (p < 0.01; Fig. 1A,B). In DN, MSTN immunostaining was observed in glomerular cells (Fig. 2C,D), mainly in podocytes (Fig. 2C), and in tubular cells (Fig. 2D–F). In addition, the MSTN signal was observed in arterial vessels (Fig. 2G,H) and, particularly, in vascular smooth muscle cells (VSMC-Fig. 2H,I).

### MSTN is expressed in tubulointerstitial infiltrates in DN

**Figure 3:**
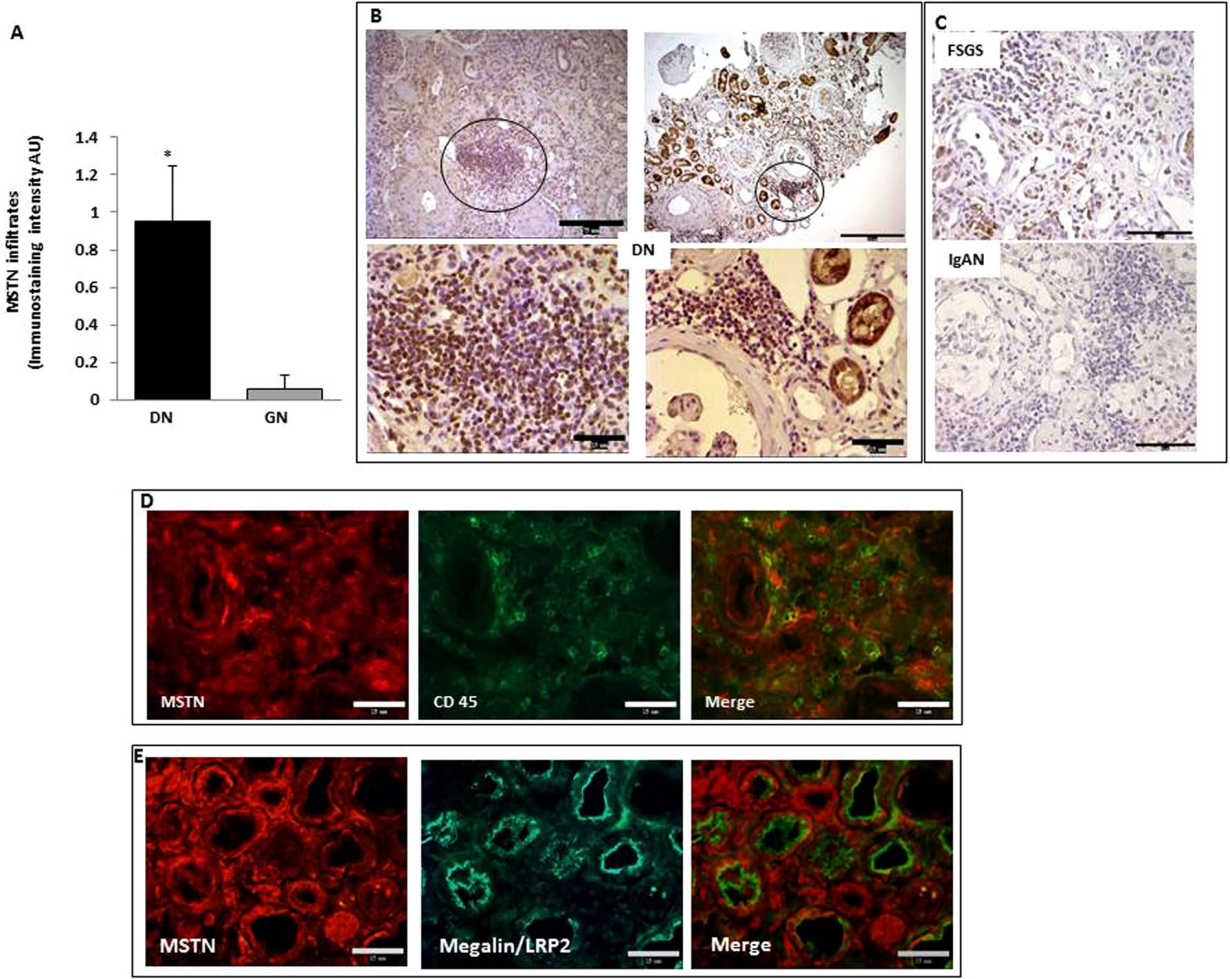
MSTN expression in infiltrating cells in DN and IgA/FSGS. (**A**) The graph depicts the levels of MSTN staining evaluated by immunohistochemistry and image analysis. Data are expressed as arbitrary units and as mean ± SEM (*p < 0.05 vs IgA/FSGS). (**B**) The photos are representative of MSTN expression in infiltrates in DN and (**C**) in IgA/FSGS biopsies. (**D**) Double staining with MSTN (red) and CD45 (green) in DN. Merged image shows that MSTN colocalizes with CD45. Panel E shows colocalization MSTN (red)+Megalin/LRP2 (green), a marker of PTECs. MSTN = Myostatin, DN = Diabetic Nephropathy, IgA = immunoglobulin A nephropathy, FSGS = focal segmental glomerulosclerosis, AU = Arbitrary Units. Magnification. Panel B: Upper photos x100 (Bar = 75–150 µm); lower photos, left x630 and right x400 (Bar = 15 µM); Panels C, D and E x400.

Tubulointerstitial infiltration is a hallmark of progressive disease in DN^21^. In our study, tubulointerstitial infiltrates were observed in 78 ± 7% of DN and 86 ± 8% of IgA nephropathy and FSGS biopsies (p = NS). Tubulointerstitial MSTN was expressed in 44 ± 5% of DN specimens but only in 10.5 ± 2% of IgA nephropathy and FSGS samples (p < 0.01) (Fig. 3A). In DN, the MSTN staining in infiltrating cells was heavier than in glomerular diseases (Fig. 3B,C) and, as also observed in progressive atherosclerotic lesions^20^, co-localized with interstitial CD45 + cells (Fig. 3D). In tubular cells, MSTN was upregulated ~3 fold (a 3.32 ± 1.56-fold increase over controls) in DN kidneys showing no interstitial infiltration, and it was upregulated ~8 fold (a 7.89 ± 2.35 fold increase over controls) in the presence of tubulointerstitial infiltrates. Figure 3E shows colocalization of Megalin/LRP2 and MSTN in the proximal tubules of a DN biopsy, whereas in control kidney only Megalin/LRP2 is detectable (Supplementary Fig. S1A).

### Activin-type IIB receptor is upregulated in the fibrotic tubulointerstitial areas and in tubulointerstitial infiltrates in DN

**Figure 4:**
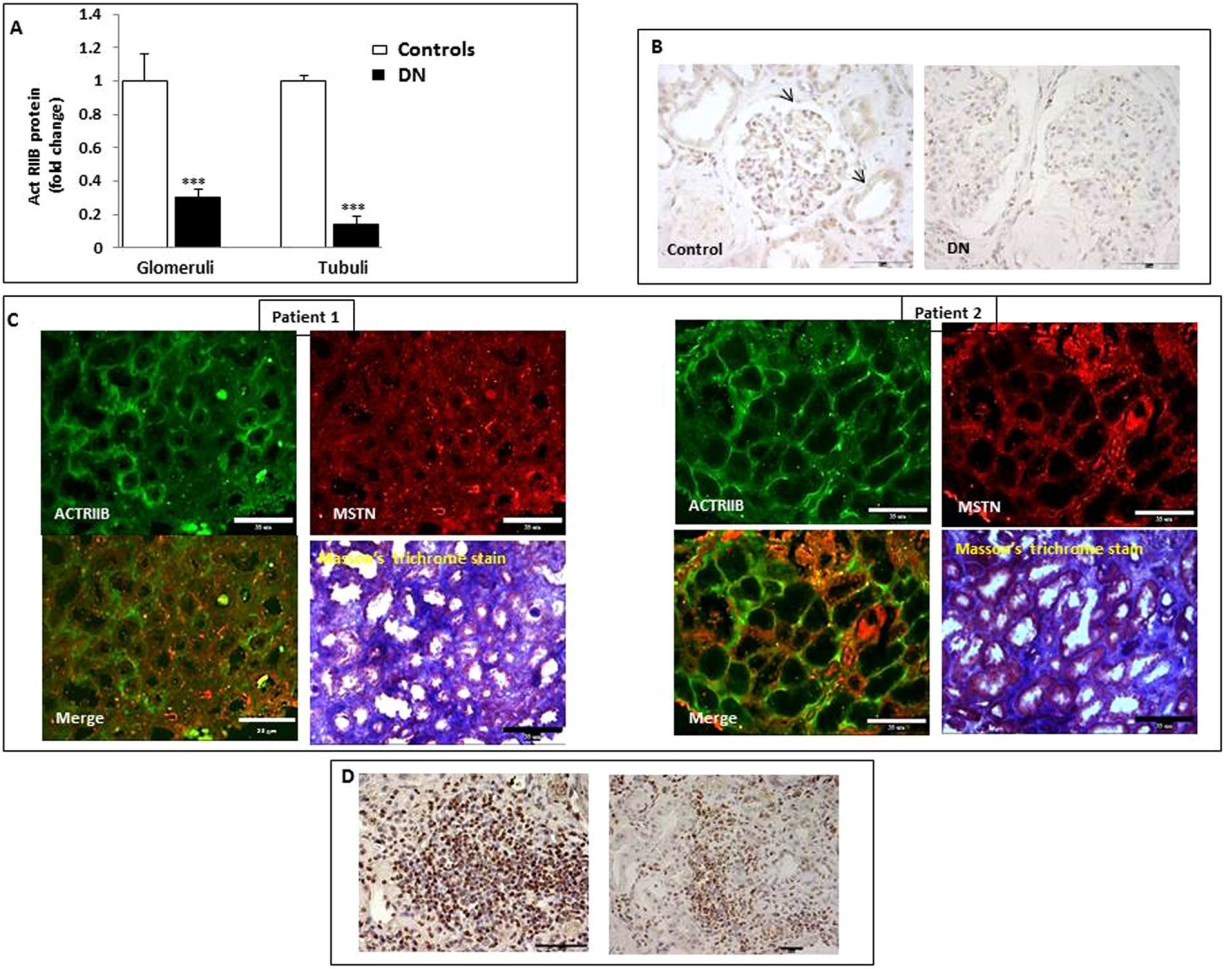
ACTR IIB expression in controls (controls) and patients with (DN). In panel A are shown the levels of ACTR IIB expressed as immunostaining intensity (fold increase), as evaluated by immunohistochemistry and image analysis (*p < 0.05 vs C). (**B**) The pictures depict ACTR IIB expression in Controls and DN sections. Arrows point out positive cells. (**C**) Mutual expression of MSTN and ACTR IIB in sections from DN patients. Interstitial fibrosis detected by Masson’s trichrome stain. (**D**) ACTR IIB expression in infiltrating cells in DN. ACTR IIB = Activin Receptor IIB, MSTN = Myostatin, DN = Diabetic Nephropathy, AU = Arbitrary Units. Magnifications: panel B x200, right x1000; panel C x200; panel D x1000. x200 Bar = 35 µm; x1000 Bar = 15 µm.

MSTN binds to Act RIIB in skeletal muscle and adipose tissue^6^. Act RIIB was expressed in both glomeruli and tubuli of kidneys in control subjects (Fig. 4A,B). In DN, Act RIIB immunostaining was downregulated when the values were collectively evaluated (Fig. 4A,B). However, Act RIIB was expressed in fibrotic areas along with upregulated MSTN (Fig. 4C). Of note, infiltrating cells were highly positive for Act RIIB (Fig. 4D). Therefore in DN ActRIIB is expressed not only in tubular cells (at a lower intensity as compared to normal kidney), but also in interstitial areas.

### MSTN expression correlates with glomerulosclerosis and interstitial fibrosis in DN

When MSTN immunostaining was related to individual structural kidney changes (Table 1), we observed that glomerular logMSTN expression was directly associated with glomerulosclerosis (Spearman’s *R* = 0.61, *p* < 0.002). In addition, logMSTN immunostaining in the tubulointerstitium was directly associated with interstitial fibrosis (*R* = 0.54, *p* < 0.01) and interstitial inflammation (*R* = 0.40, *p* < 0.05).

**Table 1:** Associations between structural changes and Logmyostatin expression in patients with type 2 diabetic nephropathy (n = 26).

*Histopathologic change*	Glomerular myostatin		Tubular myostatin		Interstitial myostatin	
	*r*	*P*	*r*	*P*	*r*	*P*
Mesangial expansion	−0.20	NS	−0.23	NS	−0.27	NS
Glomerular sclerosis %	0.61	0.01	0.099	NS	0.38	NS
Glomerular ischemia	0.13	NS	0.17	NS	−0.05	NS
Arteriolar hyalinosis	0.38	NS	−0.20	NS	0.15	NS
Interstitial fibrosis	0.29	NS	−0.125	NS	0.54	0.01
Interstitial inflammation	0.62	0.01	−0.01	NS	0.40	0.05
Tubular atrophy	0.29	NS	−0.136	NS	0.34	NS
Atherosclerosis	0.39	NS	0.17	NS	0.29	NS
Arterial hyalinosis	−0.16	NS	0.11	NS	0.07	NS

### Clinical determinants of kidney MSTN expression

As a next step, we studied whether MSTN expression in the diabetic kidney could be predicted by clinical findings. Table 2 shows the associations between individual clinical data and logMSTN expression in DN. LogMSTN expression in the glomeruli, tubuli and tubulointerstitium was directly related to serum C-reactive protein, but not to proteinuria, nor to eGFR.

**Table 2:** Clinical determinants of kidney myostatin expression in patients with type 2 diabetic nephropathy (n = 22).

Biochemical variable	Glomerular myostatin		Tubular myostatin		Interstitial myostatin	
	*r*	*p*	*r*	*p*	*r*	*p*
Creatinine (mg/dl)	0.35	NS	0.2	NS	0.13	NS
eGFR (ml/min.1.73m^2^)	−0.17	NS	−0.16	NS	−0.32	NS
Urea (mg/dl)	0.05	NS	0.02	NS	0.06	NS
Uric acid (mg/dl)	0.09	NS	0.01	NS	0.003	NS
C-Reactive Protein (mg/L)	0.88	0.01	0.61	0.02	0.72	0.01
Blood glucose (mg/dl)	0.01	NS	0.14	NS	0.04	NS
HbA1c %	0.01	NS	0.14	NS	0.02	NS
Duration of diabetes (mo.)	0.23	NS	0.17	NS	0.07	NS
Fibrinogen (mg/dl)	0.74	0.01	0.15	NS	0.11	NS
Proteinuria (g/24 h)	0.24	NS	0.03	NS	0.06	NS

### MSTN decreases HK-2 cell proliferation rate

In analogy with the MSTN inhibitory effect on cell growth and differentiation shown in skeletal muscle cells, we studied if MSTN in PTECs can negatively act on proliferation. A 24-hour MSTN treatment showed a trend in slow cell-cycle progression (control, 45 ± 3%; MSTN, 48.5 ± 1.8%; *p* = NS; Supplementary Fig. S2A). The HK-2 proliferation rate was significantly reduced after a 48-hour MSTN treatment (Supplementary Fig. S2B,C). This observation is consistent with an upregulation of P16^ink4a^ at the same time point, as shown in Supplementary Fig. 3A. No differences in apoptosis rate were observed (Supplementary Fig. S3B).

### MSTN enhances NF-κB expression in HK-2 cells

**Figure 5:**
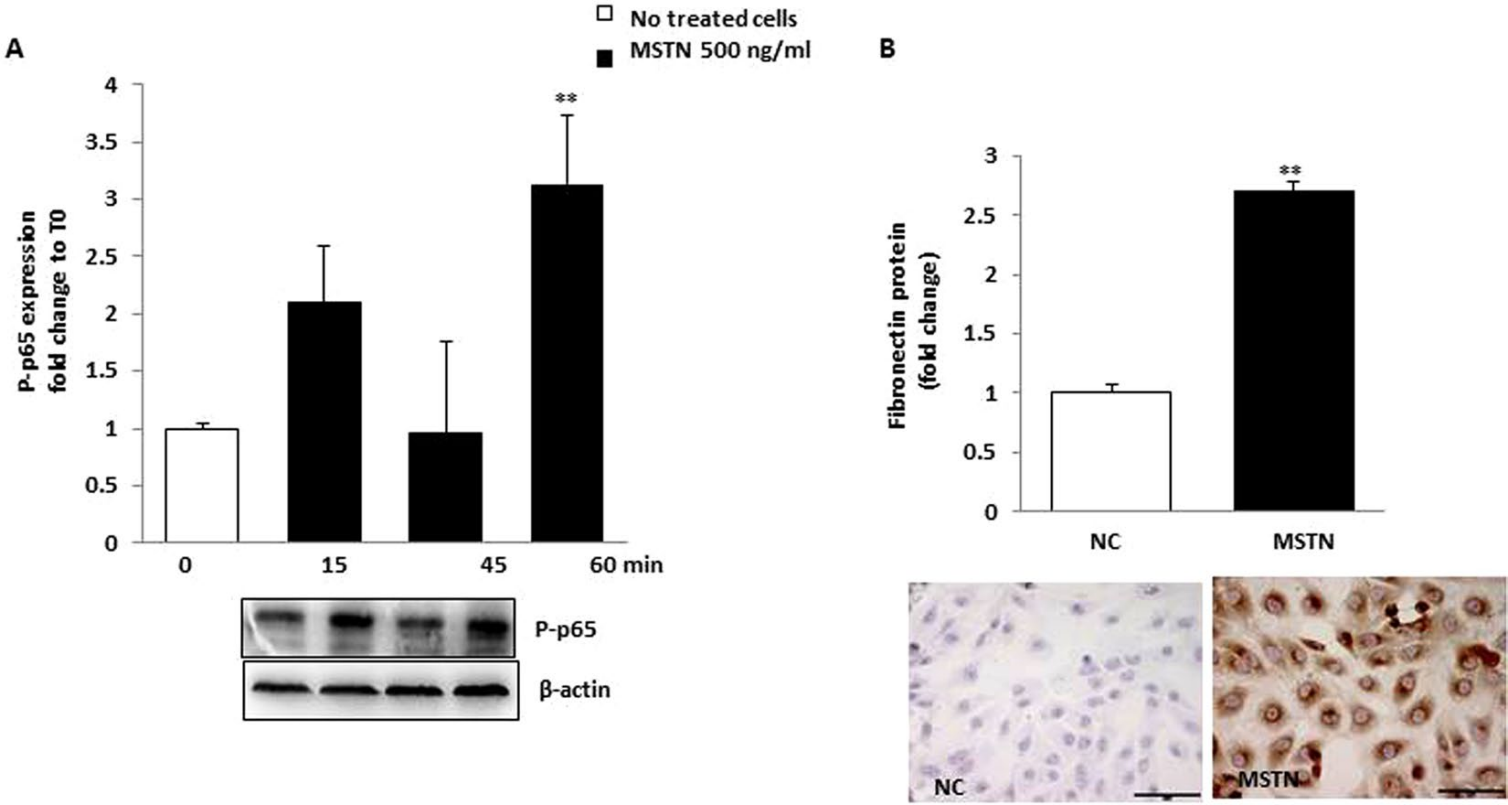
The effect of MSTN on NF-kB p65 phosphorylation and FN expression. (**A**) HK-2 were incubated for 60 minutes and p-p65 was evaluated by western blot. Blots were stripped and reprobed with anti-β actin antibody. Representative immunoblots from 3 different experiments are shown. MSTN induced NF-kB p65 phosphorylation after a 15-minute exposition period. Data are expressed as fold change ± SEM to basal value and are from 3 independent experiments (**p < 0.01 vs. T0). (**B**) HK-2 were exposed to MSTN for 48 hours and FN protein was studied by immunocytochemistry with image analysis. MSTN induced FN protein expression (by ~3 folds vs. NC, p < 0.01). Data are expressed as fold change with respect to NC. MSTN = Myostatin; P-p65 =phosphorylated p65; FN = fibronectin, NC = No treated cells Magnification x400, Bar = 35 µM.

NF-κB is a well-recognized downward MSTN effector^22,23^. As a next step we studied whether MSTN upregulates the expression of NF-kB p65 (phosphorylated p65 subunit) (p-p65). As depicted in Fig. 5A, MSTN induced NF-kB p65 phosphorylation after a 15-minute exposition period, an effect which lasted until 60 minutes.

### MSTN upregulates fibronectin and Smad2, 3 phosphorylation in HK-2 cells

MSTN induces fibrosis in skeletal and cardiac muscle^24,25^. In HK-2 cells, we studied the effects of MSTN on the expression of fibronectin, a major component of extracellular matrix. MSTN induced both fibronectin mRNA (Supplementary Fig. S5) and protein expression (Fig. 5B) as well as SMAD2,3 phosphorylation (Supplementary Fig. S4). Phospho(P)-SMAD2 peaked at 60 minutes, while phospho(P)-SMAD3 decayed (Supplementary Fig. S4).

### Effects of MSTN on ROS production

**Figure 6:**
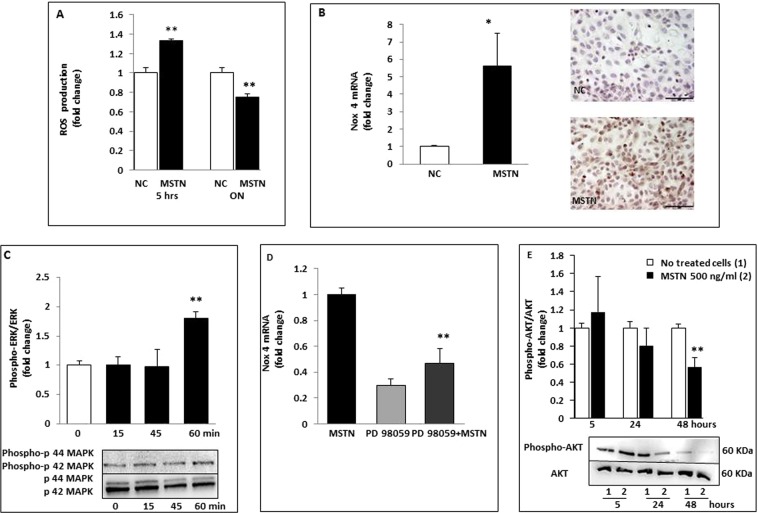
Effects of MSTN on cell signaling in HK-2 cells. (**A**) ROS production was measured by DCFH-DA staining and cytofluorimetric reading in HK-2 exposed to MSTN for 5 hours and overnight. Intracellular ROS increased by∼1.5 folds after 5-hour stimulation with MSTN (p < 0.01) and quenched after prolonged exposition (−25% with respect to NC). Values are expressed as fold change ± SEM to no treated cells and are means of 3 independent experiments. (**B**) Nox 4 expression was evaluated by rt-PCR and immunocytochemistry. Both Nox 4 mRNA and its protein were markedly upregulated (by ∼six folds, p < 0.05 vs. NC) by MSTN. (**C**) HK-2 were treated for different time intervals (0–60 minutes) with MSTN. Phosphorylated p44/42 MAPKs was detected by Western blot. MSTN induced p44/42 (ERK1/2) phosphorylation (p < 0.01 vs basal value T0).The graph represents relative phospho-p44/42 protein abundance normalized to p44/42 and data are expressed as fold change respect to T0. Immunoblots are representative from 3 different experiments. (**D**) Effects of PD 98059 (p44/42 MAPK inhibitor) on Nox 4 mRNA in HK-2 exposed to MSTN. Data are expressed as fold change respect to MSTN treated cells and as means of 3 separate experiments. (**E**) We studied the effects of ERK inhibition by PD 98059 on Nox 4 expression. Nox 4 mRNA was blunted by ERK inhibition (−50% with respect to MSTN-treated cells p < 0.01) suggesting a role of ERK in regulating ROS production by NADPH oxidase. (**E**) A 48-hour exposition to MSTN promoted phospho-AKT suppression (−40% respect to untreated cells, p < 0.01). The graph depicts phospho-AKT levels normalized to AKT and data are expressed as fold change respect to no treated cells. Immunoblots are representative of 3 different experiments. *p < 0.05, **p < 0.01. Magnification x400. Bar = 35 µM. MSTN = Myostatin; NC = No treated cells; Nox 4 = NADPH oxidase 4; rt-PCR = real-time PCR; MAPK = Mitogen-Activated Protein Kinase; ERK = extracellular signal–regulated kinases; AKT = Protein Kinase B; ROS = Reactive Oxygen Species; DCFH-DA = 2′,7′-Dichlorofluorescin diacetate.

Recently, it has been shown that MSTN is a pro-oxidant and causes ROS generation in muscle cells via the NADPH system^26^. CellRox was used to investigate the effects of MSTN on ROS production in HK-2 cells. Cytofluorimetric analysis showed that intracellular ROS increased ∼1.5-fold after 5-hour stimulation with MSTN (*p* < 0.01; Fig. 6A) and quenched after prolonged exposure (−25% with respect to untreated cells). These data indicate that MSTN can immediately induce intracellular ROS production in HK-2. To further investigate the mechanisms underlying the induction of ROS release, we examined the effects of MSTN on the expression of Nox4, a member of the renal nicotinamide adenine dinucleotide phosphate reduced form (NADPH) oxidase, and a major source of oxidative stress in DN. As shown in Fig. 6B, both Nox4 mRNA and its protein were markedly upregulated (by ∼six-folds, *p* < 0.05 vs controls) following stimulation with MSTN. As a next step, we investigated the downstream pathways involved in MSTN signaling. We found that MSTN induced p44/42 (ERK1/2) phosphorylation (*p* < 0.01 vs basal T0; Fig. 6C), while p38 and JNK were unchanged (data not shown). When we studied the effects of ERK inhibition by PD 98059 on fibronectin, cytokine, and Nox4 mRNA expression, we observed that only Nox4 mRNA was blunted (Fig. 6D; −50% with respect to MSTN-treated cells, *p* < 0.01) suggesting a role for ERK in regulating ROS production by NADPH oxidase. Lastly, in renal tubular cells, as in skeletal and adipose tissue, 48-hour exposure to MSTN promoted pAKT suppression (−40% with respect to untreated cells, *p* < 0.01; Fig. 6E).

### MSTN is upregulated by the diabetic milieu in HK-2 cells

**Figure 7:**
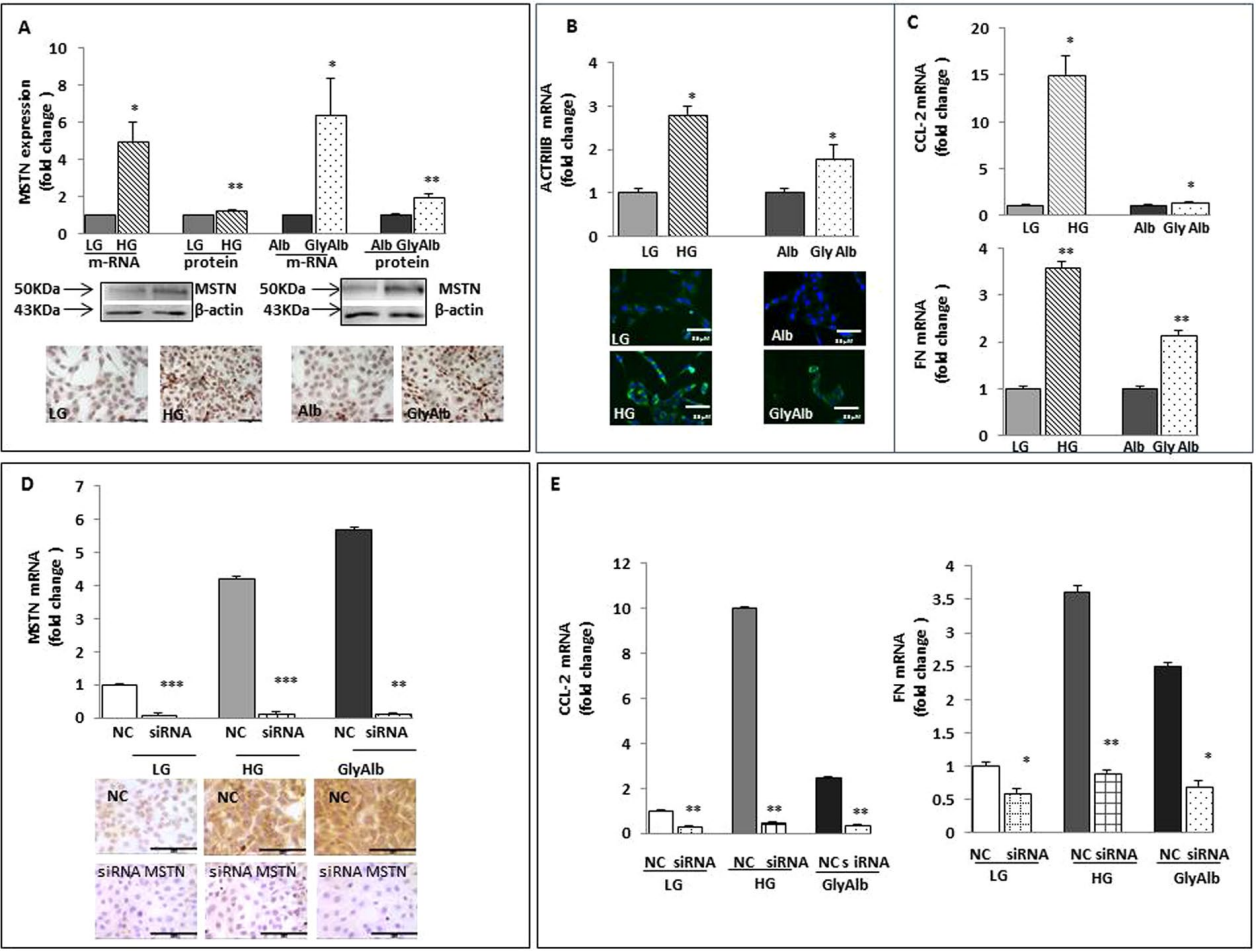
Effects of diabetic milieu on MSTN, its receptor Act RIIB, CCL-2 and FN and of MSTN silencing. (**A**) HK-2 were exposed to LG or HG, Alb or Gly-Alb for 48 hours and MSTN mRNA was measured by rt-PCR and its protein by western blot. After 48 hours, HG upregulated both MSTN mRNA and its protein (by ∼6 and ∼1.3 folds, respectively p < 0.05–0.01 HG *vs*. LG). Also Gly-Alb (500 ng/ml) increased either MSTN mRNA and protein expression (by ∼6.4 and ∼3 folds, respectively p < 0.05–0.01 vs Alb). Photos are representative of MSTN expression. Each experiment was carried out 3 times. (**B**) The graph shows Act RIIB mRNA levels, measured by rt-PCR in cells exposed to HG or Gly-Alb. The diabetic milieu increased either Act RIIB mRNA and protein expression. Pictures are representative of Act RIIB staining by immunofluorescence. (**C**) HG and Gly-Alb induced CCL-2 and FN mRNAs. Values are expressed as fold change ± SEM to LG or Alb. *p < 0.05, **p < 0.01. (**D**) Efficacy of MSTN knockdown. HK-2 were transfected with 60 nM nonspecific negative control siRNA (NC siRNA) or MSTN-specific siRNA. Gene expression was evaluated by rt-PCR after 48 hours and photos are representative of MSTN protein suppression by MSTN specific siRNA. MSTN siRNA decreased MSTN mRNA and protein in HK-2. (**E**) Downregulation of MSTN by RNA interference decreased CCL-2 and FN mRNAs *p < 0.05, **p < 0.01, ***p < 0.001 vs NC siRNA in LG, HG, Gly-Alb treated cells. MSTN = Myostatin, Act RIIB = Activin Receptor IIB; CCL-2 = Chemokine (C-C motif) ligand 2; FN = Fibronectin; LG = Low Glucose (5 mmol/L), HG = High Glucose (30 mmol/L); Alb = Albumin; GlyAlb=Glycated Albumin; rt-PCR = real time PCR. Panels A, B and D: Magnification x400. Bar = 35 µM.

To identify the triggers by which diabetes can upregulate MSTN in the kidney, we grew HK-2 cells in normal (5 mmol; NG) or high (30 mmol) glucose (HG). After 48 hours, HG upregulated both MSTN mRNA and its protein (∼6- and ∼1.3-fold, respectively, *p* < 0.05–0.01, HG vs LG; Fig. 7A). As shown in Fig. 7A, glycated albumin (GlyAlb; 500 µg/ml) also increased MSTN mRNA and protein expression (∼6.4- and ∼3-fold, respectively, *p* < 0.05–0.01). Similar results were obtained when we analyzed the effects of HG or GlyAlb on Act RIIB mRNA and protein expression (Fig. 7B). Taken together, these data show that the diabetic milieu upregulates MSTN in renal tubular epithelial cells. In addition, HG and GlyAlb induced CCL-2 and fibronectin mRNAs (Fig. 7C). As a next step, we employed MSTN silencing to examine the role of the diabetic milieu on MSTN regulation in PTECs. MSTN siRNA decreased MSTN mRNA and protein in HK-2 (Fig. 7D). MSTN gene silencing reduced MSTN-induced CCL-2 and fibronectin expression signaling (Fig. 7E).

## Discussion

MSTN affects multiple pathways of glucose and protein metabolism, yet its role in the human kidney has not been studied so far. Three issues are addressed in this study, which bear discussion. The first is MSTN expression in the human kidney, its upregulation in type 2 DN, and its association with glomerulosclerosis, interstitial inflammation and fibrosis, findings that collectively suggest that MSTN, similar to other members of the TGF-β super family, is involved in kidney fibrogenesis. The second is the proinflammatory and profibrotic action that MSTN exerts on kidney tubular cells, an effect that is similar to that previously shown in skeletal muscle. The third is the upregulation of MSTN by the diabetic milieu in proximal tubular cells, which suggests that the MSTN response in native kidney cells is a feature of the upregulated innate immunity in DN.

The overexpression of MSTN in human DN is a novel finding. In our study, MSTN was expressed in both infiltrating tubulointerstitial cells and native kidney cells; furthermore, MSTN expression was related to glomerulosclerosis and tubulointerstitial fibrosis, observations that suggest a role for MSTN in mechanisms mediating CKD. Of note, the MSTN Act RIIB receptor was downregulated in kidney tubules with normal morphology, suggesting an adaptive response to raised MSTN exposure. However, concurrent with MSTN upregulation, Act RIIB was expressed in areas of atrophy/fibrosis and in inflammatory infiltrates. Therefore in DN ActRIIB is expressed not only in tubular cells (at a lower intensity as compared to normal kidney), but also in interstitial areas.

These observations suggest that MSTN in the DN kidney acts mainly in the tubulointerstitium and in inflammatory/tubular atrophy lesions, by acting locally and/or by boosting chemiotaxis.

Both immunological and inflammatory mediators play a role in initiating and extending glomerular and tubular damage in DN^27^. We previously observed that innate immunity is upregulated in native kidney cells already at the stage of diabetic microalbuminuria^21^; at a more advanced stage, tubulointerstitial kidney cell infiltration is associated with albuminuria and fibrosis^21^, suggesting that kidney infiltration from circulating cells can accelerate tissue damage. Tubulointerstitial injury is a major feature of DN and an important predictor of renal dysfunction^28^. Several cell types, including leukocytes, monocytes, and macrophages^29,30^, are implicated in processes related to DN. In our study, MSTN was expressed more in tubulointerstitial infiltrating cells in diabetic kidney disease compared to nondiabetic proteinuric diseases, suggesting that the kidney MSTN response is induced by diabetes *per se*. In several tissues, the MSTN response is a component of the innate immune response to endogenous signals, such as free radicals^4^ and high interleukin-6^31,32^. In addition, in high-fat-induced obesity, MSTN is overexpressed in leucocytes and spleen, suggesting its role as a mediator of inflammation^32^.

In a previous study, we observed MSTN expression in infiltrating cells in atherosclerotic aorta. Colocalization studies showed that these cells expressed CD45, a marker of hematopoietic lineage. In a monocyte cell line (THP-1 cells) and in freshly isolated human monocytes, MSTN increased CCL-2 and α-SMA mRNA expression^20^. In turn, monocytes stimulated with CCL-2 displayed increased MSTN gene expression, suggesting that MSTN participates in a feed-forward inflammatory loop. Monocyte CCR-2 membrane expression was also significantly upregulated following MSTN treatment. In THP-1 cells, MSTN also acted as a chemoattractant^20^. Taken together, these data indicate that in circulating CD45+ cells and monocytes, MSTN plays an inflammatory and chemoattractive role.

The reason(s) why MSTN is more upregulated in diabetic infiltrates that in other glomerular diseases is not completely understood. In analogy with the finding that high IL-6 upregulates MSTN in muscle, diabetes-induced low grade inflammation might upregulate MSTN in circulating cells. In our study, kidney MSTN expression was neither related to the diabetes duration nor to HbA1c, while it was directly associated with serum C-reactive protein (CRP) levels.

A major question is whether the upregulation of MSTN in DN derives from MSTN + /CD45 + infiltrating cells or by MSTN overexpression by native kidney cells. In our study MSTN was already markedly upregulated (by about 3 folds) in tubules showing no interstitial infiltration, while it was even more expressed (by about 8 folds) in the presence of tubulointerstial infiltrates. In addition, high glucose was able per se to upregulate MSTN in HK-2 cells. Taken together, our data suggest that both cell infiltration and native kidney cells response contribute to abnormal MSTN expression in DN.

In our study, MSTN expression in the renal tubulointerstitium correlated with cell infiltration, in accordance with MSTN role in mediating cell recruitment. In addition MSTN in the tubulointerstitium, but not in tubuli, correlated with interstitial fibrosis. This finding suggests that MSTN overexpression in infiltrating cells, but not in tubule cells, play a role in kidney fibrosis. As a matter of fact the downregulation of Act RIIB in kidney tubules might have conferred protection from high MSTN levels. Such an effect has been observed in different cell types, when an excessive Activin/MSTN ligand level causes the internalization and degradation of Act RI/II receptor^33^,

There is increasing evidence that the immune activation in kidney cells in response to hyperglycemia or to other endogenous ligands that are upregulated by diabetes plays a major role in tissue damage^21,27^. To demonstrate the contribution of kidney tubules to MSTN overexpression, we used a human PTEC culture system (HK-2). We showed that both high glucose and glycated albumin caused an overexpression of MSTN and its receptor in human proximal tubular cells, suggesting that the hyperglycemic milieu *per se* or downward signals produced by hyperglycemia induce tubular MSTN. To recognize the transcriptional pathways that are activated by MSTN in the kidney, we studied the expression profiles of selected MSTN downward genes. In HK-2 cells, MSTN caused a decrease in replication and enhanced NF-κB activation and enrichment of several members of the NF-κB inflammatory pathway. In addition, exposure of tubular cells to glucose or glycated albumin upregulated MSTN, CCL-2, and fibronectin, effects that were blunted by MSTN silencing. All together, our findings support the hypothesis that the diabetic milieu increases MSTN production by renal cells, which results in pro-inflammatory and profibrotic effects. This is a new mechanism, linking hyperglycemia and MSTN in the pathogenesis of diabetic nephropathy.

Our findings also have other implications for the mechanisms of damage in DN. The observation that in HK-2 cells MSTN enhances ROS production through NADPH oxidase suggests that MSTN may potentiate the mechanisms of injury and cell loss already known to be active in DN^34–36^. Another finding that needs discussion is that the inhibition of the MAPK-ERK cascade downregulated the MSTN-induced NOX4 upregulation, a finding in keeping with MSTN action in muscle^37^. Therefore, the inhibition of the MAPK-ERK cascade may be another strategy to blunt MSTN effects in kidney tubular cells.

The absence of association between MSTN expression and proteinuria, and the lack of altered regulation of MSTN in renal tissues of nondiabetic kidney disease suggests that the observed MSTN activation in DN was not consequence of protein excretion.

Consistent with prior work in atherosclerotic lesions^20^, we report that MSTN was detectable in arterial vessels of patients with DN. While in leucocytes MSTN acts as a chemoattractant and increases CCL-2 dependent chemotaxis, in vascular smooth muscle cells (VSMCs) MSTN induces both cytoskeletal rearrangement and increases cell migratory rate^20^. Accordingly, our results indicate that MSTN is upregulated both in progressive abdominal aortic atherosclerosis^20^ and in the kidney vessels of patients with DN, suggesting a similar role of MSTN on vascular damage.

The present study suggests the activation of a MSTN-dependent pathway of fibrosis in DN. This hypothesis raises several issues, including a possible interaction between MSTN and other TGF-β superfamily proteins^35^. TGF-β1 and TGF-β2 have been identified as inducers of fibrosis due to their ability to recruit monocytes and myofibroblasts, activate the EMT program, and promote inflammation and apoptosis^36,37^. TGF-β mediates fibrosis *via* Smad-dependent and -independent pathways. TGF-β SMAD-independent fibrotic signaling follows activation of MEK/Erk, Rho-like GTPases, and p38 mitogen-activated protein kinase (MAPK)^36,37^. The activation of extracellular-regulated kinases (ERK) and p38 MAPK is also necessary for collagen synthesis and accumulation^37^. In our model, we observed a MSTN-induced increase in the expression of ERK and P-38 MAPK phosphorylation that may promote the development of renal fibrosis through the SMAD-independent pathway.

It is also important to consider that several activities of MSTN overlap with those of activin A, which is upregulated in mouse models of chronic kidney disease^38^. The activation of the Act RIIA in PTECs promotes apoptosis and inhibits cell growth^39^. In addition, renal interstitial fibroblasts are activated by activin A produced by tubular cells^40^.

This study has some limitations. First, we studied MSTN in patients with clinical diabetic disease. Therefore, additional work is needed to understand the time course of MSTN regulation at different stages of DN. In addition, although our data show a strong association between kidney MSTN upregulation and interstitial fibrosis, the effects of MSTN inhibition have been addressed only *in vitro*.

In summary, we demonstrated that the expression of MSTN is upregulated in both infiltrating and native kidney cells in patients with DN, and that it is associated with glomerulosclerosis and tubulointerstitial fibrosis. We observed also that MSTN decreases cell proliferation, induces the expression of NFkB, and enhances the expression of CCL-2 and fibronectin mRNA in renal proximal tubular cells. In addition, the diabetic milieu upregulates MSTN, and the blockade of MSTN signaling reduces CCL-2 and fibronectin overexpression in kidney proximal tubule cells. Our results suggest that MSTN participates in the mechanisms of kidney inflammation and is a potent inducer of proximal tubule activation in the kidney. All together, our findings suggest that MSTN overexpression contributes to kidney interstitial fibrosis in DN.

## Methods

Twenty-six patients with type 2 diabetes and albuminuria were recruited for this study from the Department of Internal Medicine, Nephrology Division, University of Genoa. The study was part of a larger study in patients with type 2 DN approved by the Ethical Committee of the Department of Internal Medicine, Genoa University^21^. The inclusion and exclusion criteria were defined to select a cohort of type 2 diabetic patients whose albuminuria was the result of DN^21^. The indications for renal biopsy were proteinuria greater than 0.5 g/d or atypical DN, and therefore, all biopsies were for clinical assessment. All subjects were informed about the nature, purposes, procedures, and possible risks of the renal biopsy before their informed consent was obtained. The procedures were in accordance with the Helsinki declaration. The clinical and laboratory characteristics of patients diabetic subjects are represented in Supplementary Table 1. Diabetic subjects (age 60 ± 4 years, 15 M/11 F) had overt DN (proteinuria 4.1 ± 2.5 g/day, eGFR= 31 ± 3 ml/min). Angiotensin-converting enzyme inhibitors and/or angiotensin receptor blockers had been withdrawn at least two weeks prior to the renal biopsy^20^.

As a control group we examined kidney tissue obtained from the healthy pole of kidneys removed because of small and localized tumors (n = 13, 8 M/5 F, 62 ± 2 yrs, eGFR= 77 ± 4 ml/min). All subjects had normal blood pressure and urinary protein excretion and were nondiabetic. To further check the specificity of MSTN expression in DN, MSTN gene and protein expressions were also studied in kidney biopsies of 22 subjects with nondiabetic proteinuria (FSGS, n = 11, IgA nephropathy, n = 11). Their demographic and clinical data are shown in Supplementary Table 1.

All kidney biopsies were analyzed by the same pathologist (G. Salvidio) who was unaware of study results. The morphological changes in kidney biopsies were considered in glass slides stained with hematoxylin and eosin, periodic acid–Schiff, trichrome and silver stain. Morphological changes, including interstitial fibrosis and tubular atrophy were classified as previously described^41^.

### Histological preparation and immunohistochemical staining

Paraffin sections (5 µm) of 2% paraformaldeyde-fixed tissue were analyzed for MSTN (Myostatin polyclonal antibody, Proteintech, LaboSpace s.r.l., Milan, Italy), its receptor Act RIIB (H-70 and G7) (Santa Cruz Biotechnology, D.B.A. Italia s.r.l., Seregno, Italy) and CD45 (Novocastra, Leica Biosystem, Milan, Italy), Immunostaining was performed as previously described^20^. MSTN protein expression was evaluated by Leica Qwin Image Analysis System (Leica, Cambridge, UK). Constant optical threshold and filter combination were set to select only the positive areas and both positive and negative tubuli or glomeruli were evaluated. For immunofluorescence, frozen tissue sections (5 µm) were fixed in cold methanol. MSTN detection was performed by Alexa Fluor® 594 Goat Anti-Rabbit IgG (Thermo Fisher Scientific, Milan, Italy) and CD45 and Act RIIB by FITC Goat Anti- Mouse IgG (Sigma Aldrich, Milan, Italy) Megalin/LRP2 was used to characterize MSTN localization in proximal tubuli. Isotype-matched antibodies corresponding with the primary antibodies were used as negative controls (Supplementary Fig. 1B). Glomeruli and the tubulointerstitial specimens were microdissected as previously described^20^. Briefly, frozen biopsy sections (7 µm thick) of kidney underwent laser capture microdissection (LCM) performed with a Veritas apparatus (Arcturus Bioscence, Mountain View, California, U.S.A.). Total RNA was extracted by Arcturus PicoPure Isolation Kit (Applied Biosystem, Life Technologies, Monza, Italy).

### cDNA RT and quantitative RT-PCR

cDNA synthesis was performed by the use of the High Capacity cDNA Reverse Transcription Kit (Applied Biosystem) and PCR amplification was carried out as previously described^20^. β-actin was used for the normalization of expression values of the other genes. Primer sequences are reported in Supplementary Table 2. Fluorescence signals measured during the amplification were considered positive if the fluorescence intensity was more than 20-fold greater than the standard deviation of the baseline fluorescence. The ΔΔ*C*T method of relative quantification was used to determine the fold change in expression^21^. Assays were run in triplicate by a Universal PCR Master Mix on Master Cycler RealPlex (Eppendorf, Hamburg, Germany) PCR system.

### Cell cultures

HK-2 cells, an immortalized proximal tubular epithelial cell line from normal adult human male kidney, were obtained from ATCC. Cells were grown in DMEM/F12 medium supplemented with 5% [v/v] FBS, 100 U/ml penicillin-streptomycin, 2 mmol L-glutamine, 5 μg/ml insulin, 5 μg/ml transferrin, 5 ng/ml sodium selenite, 5 pg/ml T3, 5 ng/ml hydrocortisone, 5 pg/ml PGE1 and 10 ng/ml epidermal growth factor. Cells were grown at 37 °C in a humidified 5% CO_2_ condition. For experiments, cells were exposed to low glucose (5.5 mmol/L) or high glucose (30 mmol/L) DMEM (Euroclone, Milan, Italy), human albumin (Sigma Aldrich) or Glycated albumin (Sigma Aldrich) (500 ng/ml), or recombinant Myostatin (0.5–1000 ng/ml) (Peprotech, LiStarFish, Cernusco S/N, Italy).

### Effects of different MSTN concentration on CCL-2, CCL-5 and Fibronectin expression on HK-2 cells

To substantiate a direct tubular contribution of MSTN overexpression in DN, we used a human proximal tubule culture system (HK-2 cells). First, to identify the most effective concentration, we exposed HK-2 to 0–1000 ng/ml MSTN for 5 hours. As depicted in Supplementary Fig. S5 (panels A, B), each concentration upregulated both CCL-2 and CCL-5 (p < 0.05–0.001). Fibronectin was upregulated by higher (50–1000 ng/ml) MSTN concentrations. Therefore, for our studies we chose the 500 ng/ml dose, which is associated with both proinflammatory and profibrotic effects in HK-2.

### Proliferation

Proliferation was evaluated by cell labelling with carboxyfluorescein succinimidyl ester (CFDA-SE; Invitrogen, Milan, Italy). Data were analyzed with the Proliferation Wizard module of the ModFit LT 4.0 software (Verity Software House, Topsham, ME, USA) and the results were expressed as Proliferation Index. Tests were performed using the Attune Acoustic Focusing Cytometer (Thermofisher Scientific, Milano, Italy).

### Oxidative stress detection

CellROX® Deep Red Reagent (Thermo Fisher Scientific) was used to detect the oxidative stress in HK-2. While reacting with ROS, this fluorogenic probe becomes brightly fluorescent. Cells were stained according to the manufacturer’s protocol and measured by Attune Acoustic Focusing Cytometer (Thermo Fisher Scientific).

### Western blot analysis

Cell layers were lysed in cold buffer (20 mM HEPES, 150 mM NaCl, 10% [v/v] glycerol, 0.5% [v/v] NP-40, 1 mM EDTA, 2.5 mM DTT, 10 ug/L aprotinin, leupeptin, pepstatin A, 1 mM PMSF, and Na_3_VO_4_). Protein concentration was determined by using the Bicinchonic Protein assay kit (Merck, Milan, Italy) and 10–20 µg were resolved on SDS-polyacrylamide gels and electro-transferred to a PVDF membrane (Merck). Blots were probed using anti Myostatin polyclonal antibody (Proteintech Europe), anti-phospho-ERK1(T202/Y204)/ERK2 (T185/Y187) (R&D Systems, Space Import-Export s.r.l., Milan, Italy), p-NFkB p65 (Ser 536) (Santa Cruz Biotechnology), anti p-SMAD2 (Cell Signaling Technology, Euroclone, Milan, Italy), anti p-SMAD 3 (Cell Signaling Technology), p16^ink4a^ (St John’s Laboratory, D.B.A. Italia s.r.l.) and reprobed with β-actin or ERK (Santa Cruz Biotechnology) or SMAD 2,3 (Cell Signaling Technology) and incubated in horseradish peroxidase secondary antibodies (Cell Signaling Technology).Immunoblots were developed with Immobilon Western chemiluminescent HRP substrate (Merck). Band intensities were determined using an Alliance system (Uvitec, Cambridge, UK).

### Immunocytochemistry and Immunofluorescence

HK-2 grown on chamber slides to sub-confluence were exposed to different stimuli as above described. After five-minute fixation in cold methanol, cells were incubated with anti-MSTN or anti-Nox-4 (Santa Cruz Biotechnology) or anti-fibronectin (Merck) antibodies. Immunostaining was performed as described previously^20,21^. Slides were counterstained with haematoxylin and examined by light microscopy.

For immunofluorescence, HK-2 were incubated with ACTR IIB (Santa Cruz Biotechnology) antibody, subsequently with FITC antibody (Merck) and then observed under the fluorescence microscopy.

### Cell mRNA analysis

HK-2 were incubated for 5 hours with or without MSTN, low (5.5 mmol/L) or high (30 mmol/L) glucose, DMEM, human albumin or glycated albumin (Sigma Aldrich) (500 ng/ml). Total RNA was isolated using the Qiazol Lysis reagent (Qiagen Italia, Milano, Italy).The RNA concentration and integrity were evaluated on a NanoDrop ND-1000 Spectrophotometer (NanoDrop Technologies Inc., Wilmington, DE, USA). 1 µg RNA was used for cDNA synthesis with iScriptcDNA synthesis kit (Biorad, Segrate, Italy). PCR amplification was carried out in a total volume of 10 μL, containing 1 μL cDNA solution, 5 μL PrecisionPLUS 2x qPCR MasterMix with Syber green (Primerdesign, Southampton, United Kingdom), 0.5 μL each primer (Primerdesign, and TIB MOL BIOL, Genoa, Italy), 3.5 μL nuclease-free water. β-actin was quantified, and used for the normalization of expression values of the other genes.

### RNA interference

HK-2 were transfected with 30 nM MSTN specific siRNA or negative control siRNA (Thermo Fisher Scientific) using Lipofectamine (Thermo Fisher Scientific) according to the manufacturer’s protocol and then incubated at 37 °C in a CO_2_ incubator for 24 hours until the cells were ready for assay. The efficacy of knockdown was determined by real-time PCR and immunocitochemistry.

### Statistical analysis

Data are presented as means ± SEM or, when skewed, median (range). The Statview statistical package (Cary, NC, USA) was used for the analysis. Means were compared for statistically significant differences by t-test, Mann-Whitney or analysis of variance when two or more than two groups, respectively, were involved. Relationships between parameters were analyzed using simple regression analysis or Spearman test, as required. A two-tailed P value < 0.05 was considered statistically significant.

## Supplementary information

Supplementary figures.

## Data Availability

The datasets generated during and/or analyzed during the current study are available from the corresponding author upon reasonable request. No applicable resources were generated or analyzed during the current study.
