# Supplementary material for: Enhanced myostatin expression and signalling promote tubulointerstitial inflammation in diabetic nephropathy
Source: Sci Rep. 2020 Apr 14;10:6343. doi: 10.1038/s41598-020-62875-2 (PMC7156449; doi:10.1038/s41598-020-62875-2)
Supplement: Supplementary file 1 — Supplementary figures. [file 41598_2020_62875_MOESM1_ESM.pdf]

Enhanced myostatin expression and signalling promote tubulointerstitial inflammation in diabetic nephropathy

Daniela Verzola[1], Samantha Milanesi[1], Francesca Viazzi[1], Francesca Ansaldo[1], Michela Saio[1], Silvano Garibaldi[2], Annalisa Carta[1], Francesca Costigliolo[1], Gennaro Salvidio[1], Chiara Barisione[2] Pasquale Esposito[1], Giacomo Garibotto[1]* and Daniela Picciotto[1]

Division of Nephrology, Dialysis and Transplantation[1] and Division of Cardiology[2] , University of Genova, Department of Internal Medicine and IRCCS Ospedale Policlinico San Martino, Genova

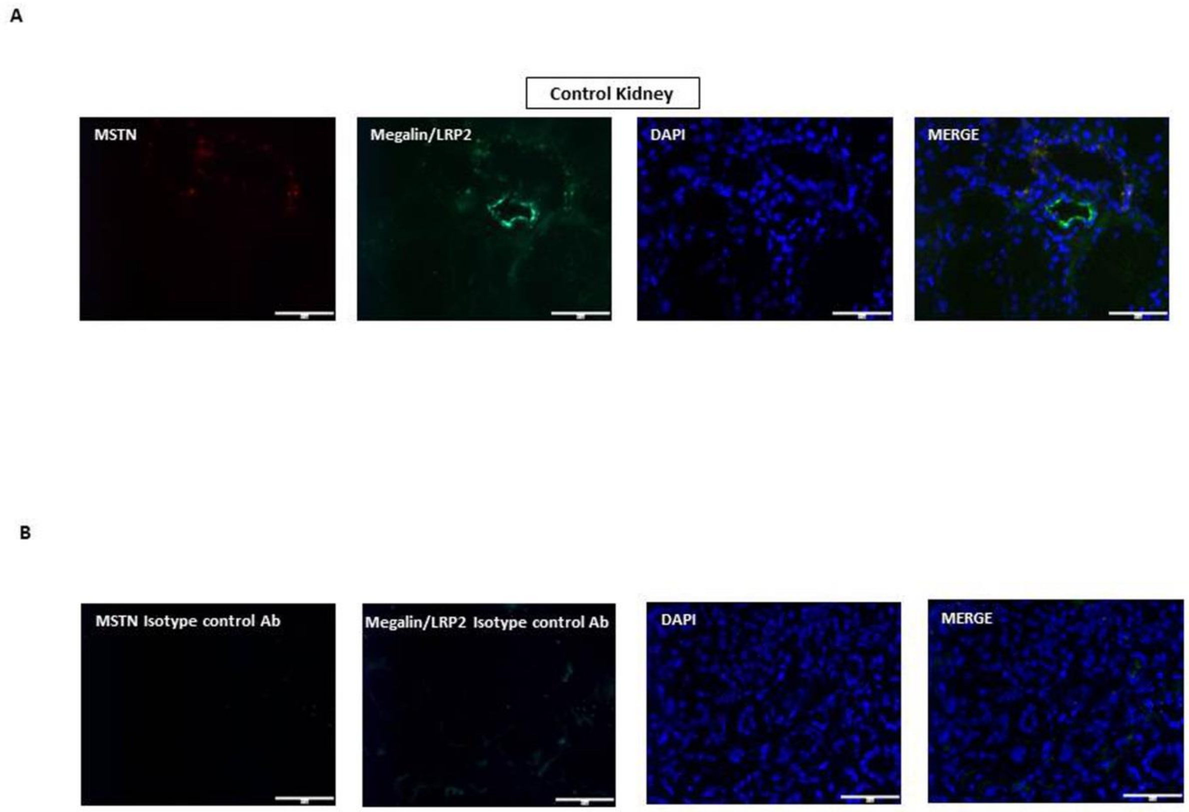

Supplementary Figure S1. MSTN expression in control kidney by immunofluorescence.

A) Colocalization of MSTN+Megalin/LRP2.

B) Isotype controls. To evaluate the level of non-specific background signal caused by anti-MSTN and /or Megalin/LRP2 antibodies, biopsies were incubated with non-immune antibodies of the same isotypes and at the same concentration as the primary antibodies, followed by incubation with secondary Alexa Fluor® 594 Goat Anti-Rabbit IgG or FITC Goat Anti- Mouse IgG.

MSTN= Myostatin, Magnification: x400 (Bar=35µM).

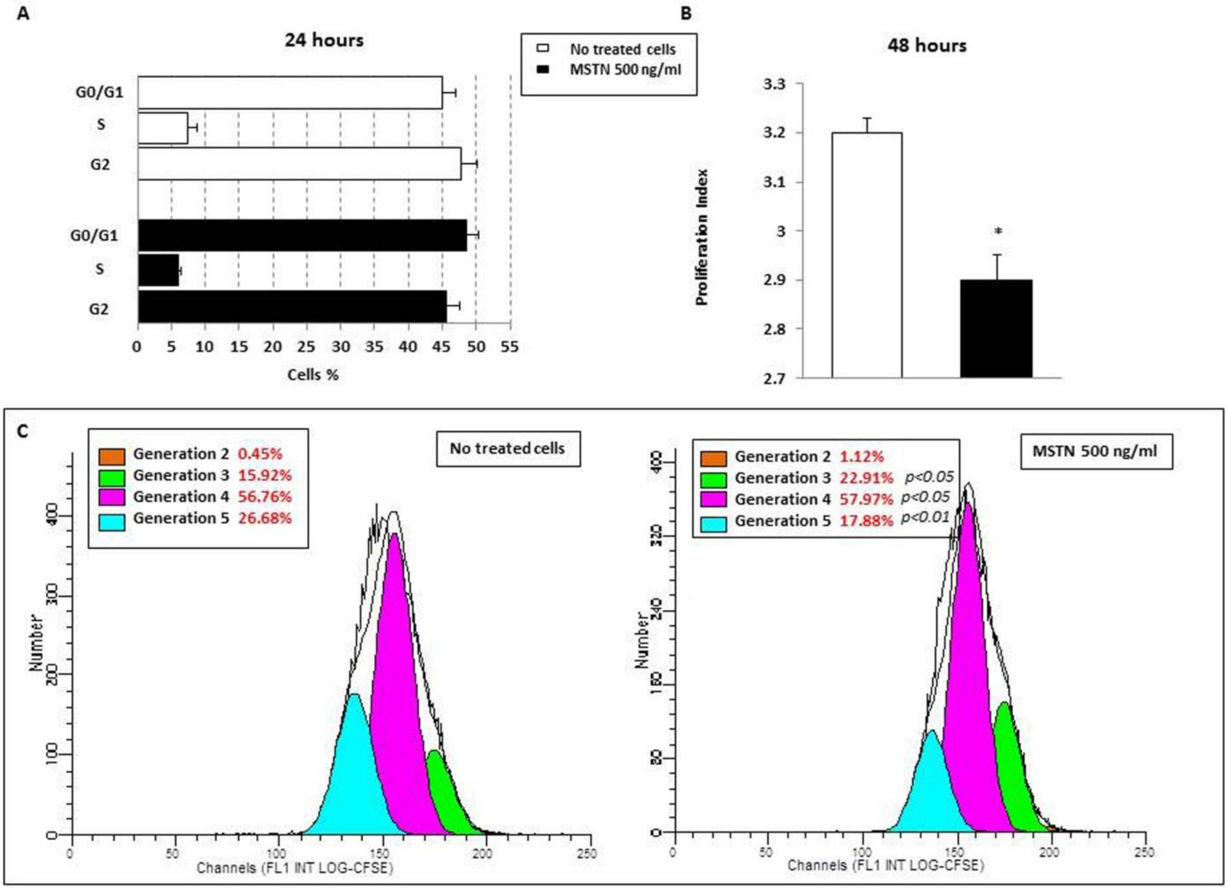

Supplementary Figure S2. Effects of MSTN on HK-2 cell cycle and proliferation index. A) 24-hour MSTN treatment lowered cell cycle as suggested by a slight increase of G1/G0 phase measured by propidium iodide staining and cytofluorimetric reading. B) At 48 hours a decreased proliferation rate is revealed by CFSE-DA assay. Values of Proliferation Index obtained on the basis of discrete peaks of progressive fluorescence halving of CFSE divided equally within two daughter cells (generation) as exemplified in Supplementary Figure 2C) . Therefore, the progressive halving fluorescence intensity is a measure of the number of cell divisions. As shown by the histograms, MSTN has a negative effect on cell proliferation: generations 2 and 3 are more fluorescent in respect to no treated cells and the percentage of the next generation 4 is strongly reduced. Data are expressed as mean ±SEM of three different experiments. (*p<0.05 vs. untreated cells).

MSTN=Myostatin; CFSE-DA Carboxyfluorescein diacetate succinimidyl ester.

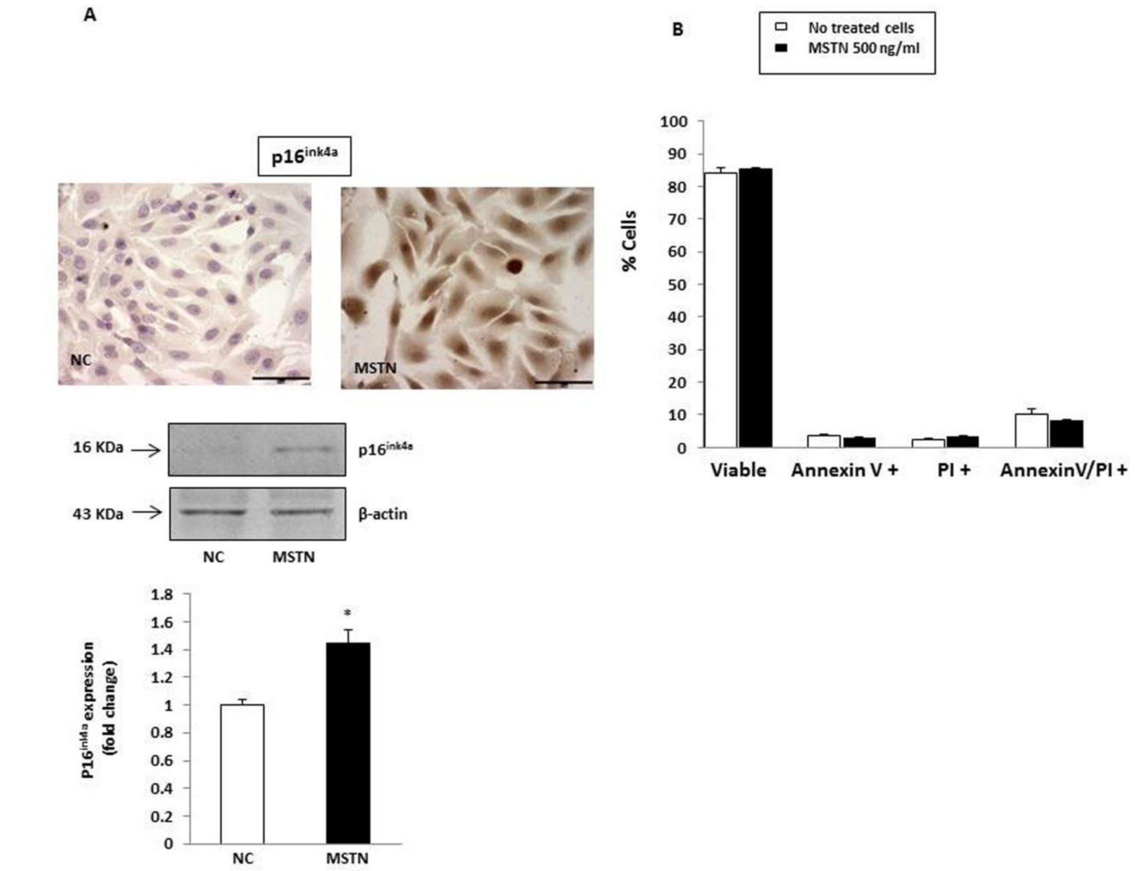

Supplementary Figure S3. A)Expression of p16ink4a by immunocytochemistry and western blot. Hk-2 were exposed to 500 ng/ml MSTN for 24 hrs. Images are representative of 3 different experiments.*p<0.05 B) Evaluation of apoptotic cells by Annexin V/PI staining and cytofluorimetric reading. No differences in apoptosis were observed.

NC=no treated cells; MSTN= Myostatin. Hrs=hours; PI=propidium iodide.

Magnification x400, Bar= 35μm

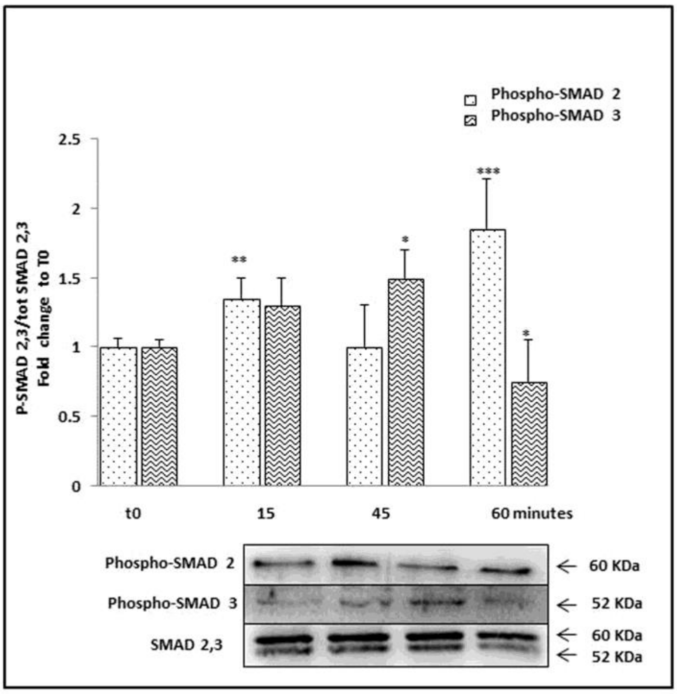

Supplementary Figure S4. Effects of MSTN on SMAD2,3 expression. HK-2 were treated for different time intervals (0–60 minutes) with 500 ng/ml MSTN. Then, phospho-SMAD2,3 were detected by Western blot. MSTN induced SMAD2,3 phosphorylation. Phospho- SMAD 2 peaked at 60 minutes, while Phospho-SMAD 3 decayed. The graph represents relative phospho-SMAD2,3 protein abundance normalized to SMAD. Data are expressed as fold change with respect to basal value (T0). Results are means ± SEM of 3 different experiments (* $p<0.05$,** $p<0.01$,*** $p<0.001$ vs. T0).

MSTN= Myostatin; SMAD= Small Mothers Against Decapentaplegic.

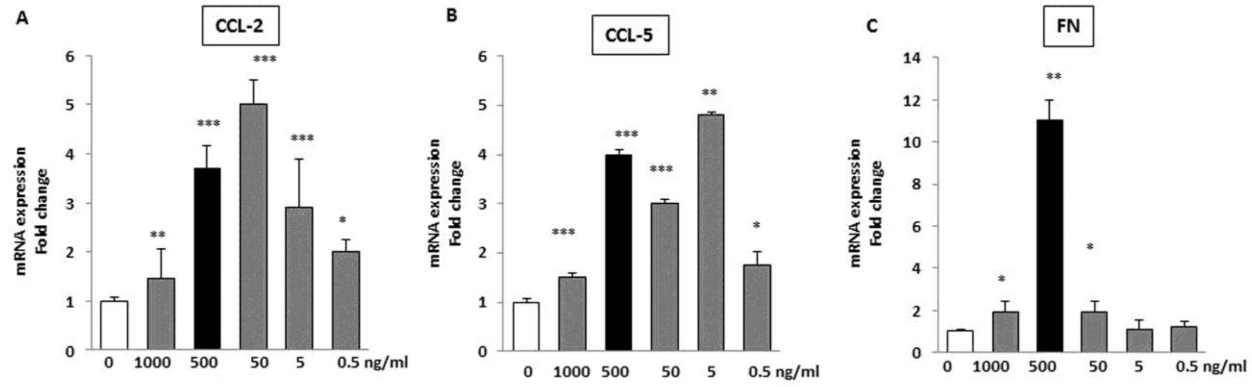

Supplementary Figure S5. Effects of increasing doses of MSTN on PTEC CCL-2 (A), CCL-5 (B), FN (C) m-RNA expression. HK-2 were exposed to 0-1000 ng/ml MSTN for 5 hours and mRNAs were studied by rt-PCR. Data are expressed as fold change with respect to basal value (T0). Results are means ± SEM of 3 different experiments (* $p<0.05$,** $p<0.01$,*** $p<0.001$ vs. T0).

PTEC= Proximal tubular cell; MSTN= Myostatin; CCL-2= Chemokine (C-C motif) ligand 2; CCL-5= Chemokine (C-C motif) ligand 5; FN= Fibronectin; rt-PCR=real-time PCR.
